# Effect of *Polygonatum cyrtonema* Flour Addition on the Rheological Properties, Gluten Structure Characteristics of the Dough and the In Vitro Digestibility of Steamed Bread

**DOI:** 10.3390/foods14234116

**Published:** 2025-12-01

**Authors:** Zhangjie Bi, Yuling Yang, Long Yang, Chao Yang, Changqing Dong, Zhipeng Liu, Zexuan Gong, Ruxin Wang, Xuebin Yin

**Affiliations:** 1 College of Food Science and Engineering, Anhui Science and Technology University, Chuzhou 233100, China; 2 Anhui Provincial Key Laboratory of Functional Agriculture and Functional Food, Chuzhou 233100, China; 3 Associated Discipline Key Laboratory of Whole Grain Nutrition and High-Value Utilization, Chuzhou 233100, China

**Keywords:** *Polygonatum cyrtonema* flour, microstructure, physicochemical properties, digestibility of steamed bread, antioxidant capacity

## Abstract

The study explores the impact of incorporating *Polygonatum cyrtonema* flour (PCF) into wheat flour on dough functionality and steamed bread quality. The results show that PCF enhanced dough hydration, rheology, and protein network stability through hydrophilic and non-covalent interactions, particularly hydrogen bonding. At the optimal level, steamed bread demonstrates improved specific volume, elasticity, and cohesiveness, accompanied by reduced hardness and chewiness, with hardness decreasing by 29%, chewiness by 25.80%, and gumminess by 26.30%. Microstructural analyses have confirmed enhanced water retention, strengthened gluten matrices, and favorable secondary structure transitions. The ultraviolet visible absorption spectroscopy and fluorescence spectroscopy analyses revealed that PCF enhanced the interactions between proteins and starch, accompanied by a red shift and decreased fluorescence intensity, indicating a more compact protein conformation. These findings suggest that PCF regulates protein secondary structures through hydrogen bonding and hydrophobic interactions, thereby stabilizing the gluten network. PCF supplementation boosted antioxidant activity and modulates starch digestibility; at a 10% substitution level, resistant starch (RS) decreases from approximately 60% in the control to 34%. This reduction indicates that PCF disrupts the integrity of the starch protein matrix, increasing amylase accessibility to starch granules and thus promoting starch hydrolysis. Incorporating 4% PCF in the formulation enhances both the technological performance and nutritional quality of the product while maintaining its overall integrity. These findings highlight the dual role of PCF in improving technological functionality and nutritional attributes. PCF emerges as a promising natural fortification ingredient for steamed bread, offering quality enhancement and additional health value.

## 1. Introduction

Steamed bread, a revered culinary heritage in Chinese cuisine with a history spanning millennia, is traditionally made from wheat flour, water, and leavening agents [1]. Despite the widespread popularity of steamed bread as a traditional Chinese staple, it is imperative to address health considerations stemming from its conventional preparation with refined wheat flour. The refining process eliminates bran and germ, leading to a notable reduction in dietary fiber, vitamins, and minerals in the final product. This nutritional composition may not adequately fulfill comprehensive human dietary requirements. Prolonged consumption of refined wheat flour as a primary dietary staple may increase susceptibility to chronic conditions such as cardiovascular diseases and diabetes. Consequently, optimizing interactions between gluten and starch is crucial for improving the textural attributes of steamed bread. Han et al. [2] characterized the microstructural properties of the gluten-starch matrix and demonstrated that supplementation with common wheat (*Triticum aestivum*) and durum wheat (*Triticum durum*) enhances dough elasticity and stability. Furthermore, researchers have explored the incorporation of sweet potato leaf powder [3] and tea polyphenols [4] to enhance the nutritional and functional profiles of steamed bread.

*Polygonatum cyrtonema*, a perennial herb of the Asparagaceae family, is widely distributed across temperate zones of the Northern Hemisphere. Documented in Shennong’s Classic of Materia Medica, it is renowned for its traditional medicinal properties in nourishing the lungs, strengthening the spleen and kidneys, and dispelling wind-dampness. With a history of over two thousand years of medicinal and culinary use in China, contemporary pharmacological investigations have revealed its principal bioactive constituents, including polysaccharides [5], saponins, flavonoids, anthraquinones [6], lignans, vitamins, alkaloids, essential amino acids, and trace elements. These compounds collectively contribute to *Polygonatum cyrtonema* documented antioxidant [7], hypoglycemic, hypolipidemic [8], anti-inflammatory [5], and anticancer properties [7]. Numerous in vivo and in vitro studies have highlighted the significant therapeutic potential of *Polygonatum cyrtonema* in the management of cardiovascular diseases [9]. Its anti-atherosclerotic, myocardial cell-protective, and myocardial fibrosis-inhibiting effects are primarily attributed to its regulation of oxidative stress, inflammatory response, and lipid metabolism pathways [8].

This study systematically quantified the pasting properties and thermodynamic behaviors of composite flours with varying blending ratios. The research investigated the mechanistic influence of incorporating *Polygonatum cyrtonema* flour on dough water distribution dynamics and quality attributes of steamed bread. The comprehensive evaluation encompassed texture profile analysis (TPA), specific volume measurement, and colorimetric analysis. Microstructural changes were characterized using scanning electron microscopy (SEM), while modifications in protein network architecture were identified through gel electrophoresis and quantification of free sulfhydryl groups. These results lay the groundwork for the innovative utilization of *Polygonatum cyrtonema* flour in flour-based products and establish a technical framework for enhancing quality through the synergistic application of food additives.

## 2. Materials and Methods

### 2.1. Materials

Wheat flour (Bainong 4199, moisture 13%, 12% protein content) was supplied by Fengbao Grain and Oil Food Group Co., Ltd. (Chuzhou, China). *Polygonatum cyrtonema* flour (moisture 3%, 11% protein content), processed through repeated steaming and drying cycles, was obtained from Jiuhuafu Jinlian Smart Agriculture Co., Ltd. (Chizhou, China). Active dry yeast was purchased from Angel Yeast Co., Ltd. (Yichang, China). All analytical-grade reagents were obtained from Solarbio Science & Technology Co., Ltd. (Beijing, China).

### 2.2. Preparation of Steamed Bread

Steamed bread was prepared following a modified method of Wang et al. [10]. Based on preliminary experiments and existing literature, substitution levels below 10% were selected to maintain desirable texture and color of the steamed buns while effectively evaluating the functional properties of *Polygonatum cyrtonema* flour. *Polygonatum cyrtonema* flour was incorporated into wheat flour at 0%, 2%, 4%, 6%, 8%, and 10% to investigate its impact on end-product quality. Both wheat flour and *Polygonatum cyrtonema* flour were sieved through a 100-mesh sieve before blending. The water absorption of each blended flour was determined using a Mixolab (Chopin Technologies, Villeneuve-la-Garenne, France) instrument, and the hydration level was set at 60% of the measured value. For each substitution level, 100 g of blended powder was used, with corresponding water additions of 60.00 g, 60.00 g, 60.40 g, 61.00 g, 61.60 g, and 62.30 g, respectively.

Flour mixtures and yeast were kneaded in a dough mixer (Supor, Hangzhou, China) for 5 min. The dough was proofed for 30 min, degassed, portioned, and proofed again for 10 min. Samples were then steamed for 25 min. The steamed bread was lyophilized (FD-103, Junde, Jinan, China), ground into powder, and stored for subsequent analysis.

### 2.3. Thermo-Mechanical Behavior of Blended Flours in the Mixolab

The Mixolab (Chopin Technologies, Villeneuve-la-Garenne, France) was utilized to assess the impact of varying proportions of *Polygonatum cyrtonema* flour on the properties of wheat dough, following the Chopin+ protocol, with minor modifications to Kim et al.’s method [11]. Wheat flour was evenly mixed with *Polygonatum cyrtonema* flour at concentrations of 0%, 2%, 4%, 6%, 8%, and 10%. Each sample was tested at its optimum water absorption. The temperature–time program was as follows: mixing at 30 °C for 8 min, heating from 30 °C to 90 °C at 4 °C/min and holding at 90 °C for 7 min, followed by cooling to 50 °C at 4 °C/min and holding at 50 °C for 5 min, with a total duration of 45 min. Torque (N·m) was recorded across five stages: C1 (maximum consistency and water absorption during dough development), C2 (protein weakening at the onset of heating), C3 (peak torque from starch gelatinization), C4 (stability of the hot paste during the high-temperature hold), and C5 (extent of starch retrogradation during cooling).

### 2.4. Thermodynamic Properties Analysis

Following the methodology established by Dang et al. [12], a precise amount of 3 mg sample and 9 mg of deionized water were weighed, transferred to an aluminum crucible, and hermetically sealed with a lid using a crimper. Subsequently, the sealed crucible was equilibrated at 4 °C for 24 h. The thermal properties of the sample were then determined using a differential scanning calorimeter (Metler-Toledo, Greifensee, Switzerland) under nitrogen protection (flow rate: 10 mL/min), with an empty crucible as a reference. The Differential Scanning Calorimetry (DSC) measurement employed a heating rate of 10 °C/min over a temperature range of 20–120 °C. Parameters such as the onset temperature (T_O_), peak temperature (T_P_), conclusion temperature (T_C_), and gelatinization enthalpy (∆*H*) were obtained through examination of the resulting thermogram.

### 2.5. Gelatinization Properties Analysis

The gelatinization properties of wheat starch were analyzed utilizing a Rapid Visco Analyzer (Nirun, Shanghai, China) with modifications based on the method [13]. A 6% (*w*/*w*) starch slurry (25 mL) was subjected to the following programmed profile: equilibration at 25 °C for 1 min, heating to 95 °C at a rate of 5 °C/min, holding at 95 °C for 10 min, cooling to 50 °C at 5 °C/min, and maintaining at 50 °C for 10 min. The rotational speed was set at 960 rpm for the initial 10 s and 160 rpm thereafter. Upon completion, the software automatically calculated parameters including Peak Viscosity (PV), Final Viscosity (FV), Breakdown (BKD), Setback (STB), Peak Time, and Pasting Temperature.

### 2.6. Microstructural Analysis of Dough

The freeze-dried dough samples were cut into small pieces, affixed to SEM (ZEISS, Oberkochen, Germany) stubs using double-sided conductive adhesive, and observed at an accelerating voltage of 3 kV and a magnification of 1000× to examine their microstructural characteristics.

### 2.7. Water Distribution Analysis

Water distribution was measured using a low-field nuclear magnetic resonance (LF-NMR) analyzer (Niumag Corporation, Suzhou, China) following a previously established protocol [14]. The parameters were set as follows: time domain (TD) = 50,000, echo time (TE) = 0.1 MS, number of echoes (C) = 2500, number of scans (NS) = 4, and relaxation delay time (T_0_) = 1500 Ms.

### 2.8. Fourier Transform Infrared (FT-IR) Spectroscopy 

The secondary structure analysis of gliadin was conducted using Fourier transform infrared (FT-IR) spectroscopy according to the method [15]. Specifically, 1 mg of gliadin was mixed with 100 mg of potassium bromide (KBr, Solarbio, Beijing, China) and ground thoroughly in an agate mortar. The mixture was compressed into a thin pellet under hydraulic pressure and analyzed using an FT-IR spectrometer (FT-IR-850, Gangdong Science and Technology, Tianjin, China). Spectra were generated by accumulating 64 cumulative scans at a resolution of 4 cm^−1^ over the range of 400–4000 cm^−1^. The amide I band (1600–1700 cm^−1^) was processed using OMNIC 8.2 software, including baseline correction, Gaussian deconvolution, second-derivative analysis, and curve fitting, to quantify the relative percentages of secondary structure components.

### 2.9. Sodium Dodecyl Sulfate–Polyacrylamide Gel Electrophoresis (SDS-PAGE)

SDS-PAGE analysis was carried out following the method described by Yang et al. [16] using a 12% separating gel (pH 8.8) and 5% stacking gel (pH 6.8). Freeze-dried dough powder containing precisely 8 mg of protein was weighed into a 2 mL centrifuge tube and mixed with 1 mL non-reducing sample buffer (0.01 mol/L Tris-HCl, pH 6.8, 10% SDS, 10% glycerol, 0.1% bromophenol blue). The mixture was then vortexed at 2000 rpm for 1 h, statically incubated for 1 h, boiled for 5 min, cooled, and centrifuged at 12,000 rpm for 10 min to collect the supernatant. Subsequently, 5 µL of the supernatant and 3 µL of protein marker (Thermo Fisher Scientific, Waltham, MA, USA; molecular weight 10–180 kDa) were added to each lane, and electrophoresis was performed using the Mini-PROTEAN Tetra Cell system (Bio-Rad, Hercules, CA, USA) at 80 V. The gel was then subjected to Coomassie Blue R-250 (Solarbio, Beijing, China) staining and imaging via a microscopy system. For the SDS-insoluble fraction, the pellet was resuspended in reducing buffer (0.05 mol/L Tris-HCl, pH 6.8, 10% SDS, 10% glycerol, 0.1% bromophenol blue, 1% dithiothreitol, all the above reagents were obtained from Solarbio, Beijing, China), followed by the same procedures as described above for analysis.

### 2.10. Free Thiol and Disulfide Bond Analysis

Measurements were performed using a microplate reader (KD-870, Cody, Shanghai, China). Free and total thiols were quantified by a modified method [17] for free thiols, 0.50 mL sample was mixed with 40 μL Ellman’s reagent (5,5′-Dithiobis 2-nitrobenzoic acid, Solarbio, Beijing, China, 4 mg/mL), incubated 30 min, centrifuged at 12,000× *g* for 10 min, and read at 412 nm; for total thiols, 0.20 mL sample was treated with 0.20 mL Dithiothreitol (DTT, Solarbio, Beijing, China) (0.5% *w*/*v*) and 1 mL Trichloroacetic acid (TCA, Macklin, Shanghai, China) (12% *w*/*v*), incubated 60 min, centrifuged at 12,000× *g* for 10 min, the pellet resuspended in 0.5 mL urea (8 M) with 80 μL Ellman’s reagent, incubated 30 min, centrifuged at 12,000× *g* for 10 min, and read at 412 nm. All samples were prepared by mixing 0.0075 g flour with 1 mL Tris-glycine-urea-guanidine HCl buffer (pH 8.0). The calculation formulas for free thiol and disulfide bond quantification are as follows:

(1) $$SH(\mu mol/g)=(73.53\times A_{412}\times D)/C$$

(2) $$Total-SH(\mu mol/g)=(73.53\times A_{412}\times D)/C$$

(3) $$SS\left(\mu mol/g\right)=(N_{2}-N_{1})/2$$

In the formula, *A*_412_ represents the absorbance value at λ = 412 nm; The 75.53 in the formula represents the molar mass of cysteine (g/mol) and is used to convert the measured absorbance into the contents of free and total sulfhydryl groups. The dilution factor *D* was set to 1.04 for free sulfhydryl groups and 2.7 for total sulfhydryl groups; *C* denotes the final protein concentration of the sample; *N*_2_ indicates the total sulfhydryl content in the protein after reduction of disulfide bonds; *N*_1_ represents the free sulfhydryl content.

### 2.11. Non-Covalent Interactions

Measurements were performed with a microplate reader (KD-870, Cody, Shanghai, China). A Bovine Serum Albumin (BSA, Solarbio, Beijing, China) standard curve was prepared (100 µg/mL stock; calibration for solutions A–D with R^2^ = 0.999), and protein content was quantified against this curve in triplicate per dough sample [17]. Protein interactions were inferred from soluble protein differences: ionic (A–B), hydrogen (B–C), and hydrophobic (C–D). For analysis, 10 mg flour was extracted with 1 mL extraction buffer, incubated for ≥2 h, and centrifuged (12,000× *g*, 10 min). Then, 20 µL supernatant was mixed with 1.0 mL filter-sterilized Coomassie Brilliant Blue G-250 (Solarbio, Beijing, China), vortexed, and 200 µL aliquots were read at 595 nm within 5–15 min. Reagents: 0.2 M phosphate buffers (Solution A: 31.21 g NaH_2_PO_4_·2H_2_O/L; Solution B: 71.64 g Na_2_HPO_4_·12H_2_O/L); 50 mM PBS, pH 7.5 (40 mL A + 210 mL B, to 1 L); Coomassie reagent (100 mg dye in 50 mL 95% ethanol + 100 mL 85% H_3_PO_4_, to 1 L with ultrapure water, filtered, light-protected); sequential extraction buffers—A: 2.922 g NaCl/L PBS; B: 35.064 g NaCl/L PBS; C: 35.064 g NaCl + 90.09 g urea/L PBS; D: 35.064 g NaCl + 480.48 g urea/L PBS.

### 2.12. UV–Vis Absorption Spectra

The ultraviolet (UV) spectra of the samples were recorded using a UV spectrophotometer (UV-2600, Shimadzu Corporation, Kyoto, Japan) within the wavelength range of 200–400 nm at room temperature [18]. A 0.02 g portion of the sample was accurately weighed and dispersed in 20 mL of 0.01 mol/L phosphate-buffered solution. Immediately after the addition of the buffer, timing was initiated, and the suspension was allowed to stand for 1.5 h. The mixture was then vortexed for 30 s and centrifuged at 4000 r/min for 10 min, after which the supernatant was collected for measurement.

### 2.13. Intrinsic Fluorescence Spectra

The intrinsic fluorescence spectra were determined following the method [18] with slight modifications. A 500 mg portion of the sample powder was accurately weighed and dissolved in 20 mL of 0.01 mol/L phosphate-buffered solution (pH 7.0) to prepare a protein solution. Then, 3 mL of the solution was transferred into a four-sided quartz cuvette, and the fluorescence spectra were recorded using a fluorescence spectrophotometer (F-7000, Hitachi High-Technologies Corporation, Tokyo, Japan). The excitation wavelength was set at 290 nm, and the emission spectra were scanned in the range of 300–400 nm.

### 2.14. Physical Properties of Steamed Bread

#### 2.14.1. Texture Profile and Specific Volume Analysis

Steamed bread samples underwent texture profile analysis (TA. XTplus, Stable Micro Systems, Surrey, UK) using a texture analyzer. After 1 h cooling post-steaming, samples were sliced into uniform 15 mm thick sections using a precision slicer (Midea, Guangzhou, China). A P/36R cylindrical probe was employed with the following parameters: Pre-test speed: 1.0 mm/s, Test speed: 0.8 mm/s, Post-test speed: 1.0 mm/s, Trigger force: 5 g (0.049 N), Strain: 50%, Inter-compression interval: 5 s.

Specific volume was determined via calibrated millet displacement. Cooled samples (1 h post-steaming) were weighed (*M*, g). A volumetric container was filled with millet, leveled, and the initial volume was recorded (*V*_1_, mL). Subsequently, after emptying the container, the sample was placed inside, covered with millet, leveled again, and the final volume was recorded (*V*_2_, mL). Specific volume (*P*, mL/g) was calculated according to the formula:

(4) $$P=(V_{1}-V_{2})/M$$

#### 2.14.2. Color Difference Analysis

The samples prepared in Section 2.2 were freeze-dried and pulverized into powder for chromaticity assessment employing an automatic colorimeter (NR110, 3NH Technology, Shenzhen, China). After three replicate measurements per sample, the average values of color parameters were determined. The measured parameters comprised *L*, *a*, and *b* values. Subsequently, the lightness (L*), redness (a*), yellowness (b*), and total color difference (∆*E*) of PCF steamed bread were calculated using the following formulas:

(5) $$\Delta E=\sqrt{{\left(\Delta L^{*}\right)}^{2}+{\left(\Delta a^{*}\right)}^{2}+{\left(\Delta b^{*}\right)}^{2}}$$

#### 2.14.3. Pore Structure Analysis

Steamed bread samples containing varied concentrations of *Polygonatum cyrtonema* flour were cut into 10 mm thick sections using a precision slicer. The textural attributes of the slices were captured with a flatbed Image scanner. A central region measuring 3 cm × 3 cm was selected from each scanned image for subsequent texture analysis using ImageJ software (version 1.53e).

### 2.15. In Vitro Antioxidant Capacity Assay

#### 2.15.1. DPPH Radical Scavenging Activity

The antioxidant capacity of steamed bread was assessed using the 2,2-diphenyl-1-picrylhydrazyl (DPPH) radical scavenging assay. According to the instructions provided with the commercial kit (Beijing Solarbio Science & Technology Co., Ltd., Beijing, China). For sample preparation, 2.00 g freeze-dried steamed bread powder (Section 2.2) was mixed with 20 mL anhydrous ethanol and extracted for 30 min at room temperature in an orbital shaker bath. The mixture was then filtered and centrifuged at 4000× *g* for 10 min, and the resulting supernatant was collected as the sample solution. A DPPH standard was prepared by dissolving 4 mg DPPH in anhydrous ethanol and bringing the volume to 100 mL in a volumetric flask. Absorbance at 517 nm was measured with a microplate reader (KD-870, Cody, Shanghai, China). The results are expressed as milligrams of Trolox equivalents per gram of dry weight of the sample (mmol TE/g DW) [19]. The formula for the free radical scavenging rate is as follows:

(6) $$Percentage\;scavenging\;of\;DPPH=\left[(A_{0}-\left(A_{1}-A_{2}\right)\right]/A_{0}\times 100$$

where *A*_0_ represents the DPPH solution mixed with anhydrous ethanol; *A*_1_ represents the sample solution mixed with the DPPH solution; and *A*_2_ represents the sample solution mixed with anhydrous ethanol.

#### 2.15.2. ABTS Radical Scavenging Activity

Antioxidant activity was evaluated using the ABTS radical scavenging assay. According to the instructions provided with the commercial kit (Beijing Solarbio Science & Technology Co., Ltd., Beijing China). The ABTS stock solution was prepared by mixing equal volumes of 2.6 mmol/L potassium persulfate and 7.4 mmol/L ABTS, incubating for 14 h at room temperature in darkness, and storing protected from light. For samples, 2.00 g freeze-dried steamed bread powder (Section 2.2) was homogenized with 20 mL anhydrous ethanol, extracted for 30 min at room temperature in a shaking water bath, and centrifuged at 4000× *g* for 10 min; the supernatant was collected. The ABTS working solution was obtained by diluting the stock with distilled water to an absorbance of 0.70 ± 0.02 at 734 nm and used promptly. Controls were: *A*_3_, ABTS solution + anhydrous ethanol; *A*_4_, sample solution + anhydrous ethanol; and *A*_5_, sample solution + ABTS solution. The results are expressed as milligrams of Trolox equivalents per gram of dry weight of the sample (mmol TE/g DW). [19]. The formula for the free radical scavenging rate is as follows:

(7) $$Percentage\;scavenging\;of\;ABTS=\left[(A_{3}-\left(A_{5}-A_{4}\right)\right]/A_{3}\times 100$$

where *A*_3_ is the ABTS solution mixed with anhydrous ethanol; *A*_4_ is the sample solution mixed with anhydrous ethanol; and *A*_5_ is the sample solution mixed with the ABTS solution.

### 2.16. In Vitro Gastrointestinal Digestion Simulation

A glucose standard curve was initially constructed with the equation y = 0.3354x + 0.032 (R^2^ = 0.999). Total starch content was quantified using a commercial assay kit (Solarbio, Beijing, China). To determine RDS, SDS, and RS, 500 mg of freeze-dried steamed bread powder was hydrolyzed enzymatically in acetate buffer (pH 5.2) at 37 °C. Samples were withdrawn at 0, 10, 20, 30, 60, 90, 120, and 180 min, treated with ethanol to terminate the reaction, and centrifuged. The supernatant was analyzed using the 3,5-dinitrosalicylic acid (DNS) method at 540 nm. Digestive enzyme solution (containing α-amylase and amyl glucosidase) and DNS reagent were prepared following standard procedures [20]. In the equation, G_0_, G_20_, and G_120_ represent the glucose content (in mg) measured from the hydrolysate sampled at 0, 20, and 120 min, respectively, while *S* denotes the total starch content (in mg) in the corresponding sample.

(8) $$RDS(\%)=\left[\left(G_{20}-G_{0}\right)\times 0.9\right]/S\times 100$$

(9) $$SDS(\%)=\left[\left(G_{120}-G_{20}\right)\times 0.9\right]/S\times 100$$

(10) RS(%)=100−RDS−SDS

The starch hydrolysis kinetics were described using a nonlinear model, with the first-order equation as follows:

(11) $$C=C_{\infty }\times (1-e^{-kt})$$

where *C* represents the starch digestibility of the sample at specified enzymatic hydrolysis time points (0, 10, 20, 30, 60, 90, 120, and 180 min), *C_∞_* denotes the equilibrium hydrolysis concentration at infinite time, *k* is the hydrolysis rate constant and *t* is the hydrolysis time.

### 2.17. Statistical Analysis

All experiments were performed in triplicate, and the results are expressed as mean ± standard deviation (SD). Statistical analysis was conducted using SPSS 27.0 software, employing one-way analysis of variance (ANOVA) followed by Duncan’s multiple range test for post hoc comparisons. Statistical significance was established at *p* < 0.05. Graphical representations were generated using Origin 2024 software.

## 3. Results

### 3.1. Polygonatum Cyrtonema Flour Effects on Wheat Flour Farinograph Properties

To analyze the key behaviors of starch, we used Mixolab curve parameters—water absorption, dough development time, and stability—to index gluten-network formation; concurrently, the gelatinization peak and retrogradation tendency were employed to delineate starch thermal transitions and aging.

**Figure 1:**
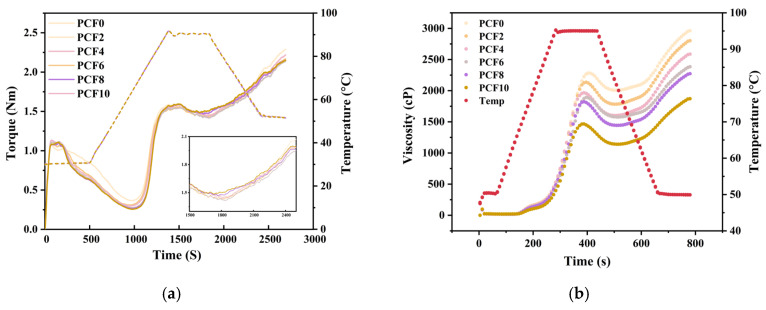
Effect of *Polygonatum cyrtonema* flour addition on the viscoelastic and gelatinization properties of wheat flour. (**a**) Thermo-mechanical behavior of blended flours in the Mixolab; (**b**) Rapid Visco Analyzer. PCF0, PCF2, PCF4, PCF6, PCF8, and PCF10 represent samples with 0%, 2%, 4%, 6%, 8%, and 10% (*w*/*w*) *Polygonatum cyrtonema* flour substitution, respectively.

Table S1 and Figure 1a revealed a notable impact of *Polygonatum cyrtonema* flour addition on the farinograph properties of the composite flour. Water absorption (WA) increased with PCF, and the gluten-building phase lengthened. In parallel, the protein-weakening rate (α) became less negative and both CS and C2 torques declined, consistent with improved tolerance to sustained mixing/thermal stress at moderate inclusion. On the starch side, C3 remained broadly comparable, whereas C4 rose slightly and C5 decreased at 6–8% PCF, indicating reduced thermal/enzymatic weakening and curtailed short-term retrogradation [21]. This phenomenon is attributed to the pronounced hydrophilic nature of *Polygonatum* polysaccharides inherent in the powder [22]. In terms of stability time, a pivotal parameter reflecting the dough’s ability to withstand mechanical mixing, the dough displayed optimal farinograph properties at a 6% inclusion level of PCF. At this concentration, both the dough development time and stability time reached their peak values, with the development time notably extended by 50% compared to the 4% inclusion group. This enhancement can be attributed to the interaction between *Polygonatum* polysaccharides and gluten components, forming a hydrophilic colloidal complex that strengthens the gluten protein network structure. Nevertheless, upon surpassing the 6% level of PCF addition, a decline in the farinograph characteristics of the dough was observed. This decline was marked by a decelerated rise in water absorption rate, coupled with approximately 3% reductions in both development time and stability time, signifying that excessive supplementation adversely affects the rheological properties of the dough.

A moderate level of PCF can densify the composite matrix without overly diluting gluten, whereas beyond the optimal addition, the benefits gradually diminish as network dilution and particle crowding become dominant.

### 3.2. Thermal Characteristic Analysis

The gelatinization temperature of starch serves as a critical indicator for evaluating the thermodynamic behavior of steamed bread. Incorporation of PCF demonstrated negligible effects on the onset temperature (T_O_) and enthalpy change (ΔH) of dough, while exerting measurable influences on peak (T_P_) and conclusion temperatures (T_C_). As shown in Table 1, at a 6% addition level, the highest T_P_ and T_C_ values were observed, representing increases of 1.5% and 2.2%, respectively, compared to the control group. This thermal shift can be attributed to alterations in starch composition (amylose-amylopectin ratio) and granule morphology within the composite flour system, wherein larger starch granules impede water penetration, leading to elevated gelatinization temperatures [13]. The modulated thermal profile signifies enhanced resistance to gelatinization degradation, indicative of improved heat stability in the gluten network. It has been proven that *Polygonatum* polysaccharides alter the gluten microstructure by forming cross-linking with gliadin-glutenin complexes, potentially redirecting reaction pathways during thermal denaturation [23].

### 3.3. Analysis of Pasting Properties 

The influence of *Polygonatum cyrtonema* flour addition on flour viscosity characteristics was quantified via RVA parameters, including peak viscosity, trough viscosity, breakdown viscosity, final viscosity, setback viscosity, peak time, and pasting temperature. Analysis of Table S2 and Figure 1b revealed that peak viscosity, trough viscosity, final viscosity, and setback viscosity all decreased as the level of PCF increased. Specifically, the sample with 6% PCF exhibited the minimum breakdown viscosity, the maximum peak time, while the pasting temperature showed minimal variation.

The reductions in both peak viscosity and trough viscosity following PCF addition suggest that PCF has a low intrinsic viscosity or behaves as a non-viscosifying component. This phenomenon is attributable to cross-linking interactions between PCF and starch molecules, leading to a compromise in the overall stability and shear resistance of the system. Setback viscosity reflects the short-term retrogradation tendency of starch. The incorporation of PCF notably reduced starch setback, with the most significant reduction (21.4% compared to the control) observed at the 6% addition level. This inhibitory effect on retrogradation arises from hydrophilic constituents in PCF attracting bound water molecules via interaction with polar groups on the starch surface [24]. This enhanced hydration enhances the stability of the starch paste and inhibits the reassociation of starch molecules.

### 3.4. Scanning Electron Microscopy

**Figure 2:**
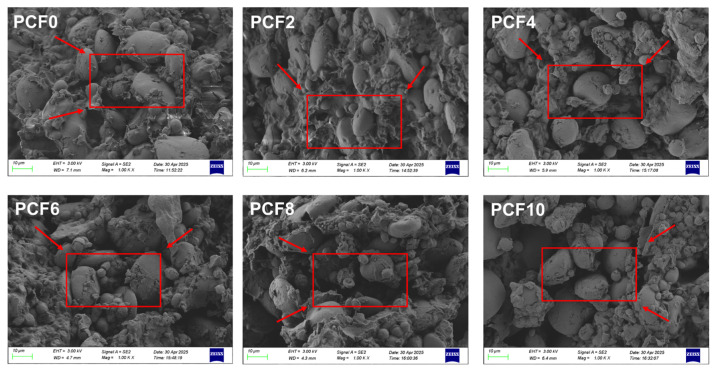
SEM micrographs of dough with increasing PCF levels. PCF0, PCF2, PCF4, PCF6, PCF8, and PCF10 represent samples with 0%, 2%, 4%, 6%, 8%, and 10% (*w*/*w*) *Polygonatum cyrtonema* flour substitution, respectively.

SEM has become an indispensable analytical tool in food science research, renowned for its exceptional high-resolution imaging capabilities and ability to provide detailed three-dimensional morphological characterization, facilitating precise examination of dough microstructure [25]. Backscattered electron scanning electron microscopy (BSE-SEM) was employed to characterize the microstructure of the dough cross-section (Figure 2). BSE-SEM analysis effectively delineated the surface morphology and compositional characteristics, revealing the presence of starch granules with diverse sizes and shapes, along with broken starch granules and partially gelatinized starch.

SEM micrographs revealed prominent, large starch granules abundantly present in the control dough (0% PCF). As the level of PCF increased, there was a significant decrease in the exposure of large starch granules on the dough surface, with the most pronounced difference in microstructure observed between the 4% addition group and the control. Enriched in polysaccharides, PCF facilitated the formation of a smoother and denser microstructure in the steamed bread, effectively encapsulating starch granules and reducing their exposure. The decline in large starch granules implies that PCF inhibits water absorption and swelling of starch granules, thereby reducing their accessible surface area for digestive enzymes and potentially enhancing the digestibility of the steamed bread. This microstructural alteration is attributed to the hydrophilic nature of PCF, which allows it to form a colloidal network upon hydration, limiting water uptake by starch granules. This colloidal network promotes physical interactions among starch, protein, lipids, and *polygonatum* polysaccharides, enhancing encapsulation and thereby reducing starch granule exposure and swelling. SEM analysis has further validated that the incorporation of PCF disrupts the establishment of a continuous gluten network and compromises its integrity, leading to alterations in dough rheological properties. The initial improvement in softness and elasticity at 4% PCF can be attributed to the denser, more uniform structure, while the textural deterioration at higher substitution levels is a direct consequence of the severely disrupted gluten network, which fails to provide the cohesive strength needed for a soft, resilient crumb. This observation is in line with prior studies indicating that the addition of PCF diminishes dough stability and viscoelasticity. These findings are also consistent with the conclusions of Li et al. [26], whose research demonstrated that the inclusion of yam flour disrupts the dough network structure, thereby influencing its viscoelastic properties.

### 3.5. Water Distribution State

Low-field nuclear magnetic resonance (LF-NMR) was employed to characterize the moisture content and binding states of water within the dough. The relaxation times t_21_, t_22_, and t_23_ reflect the molecular mobility of strongly bound water, loosely bound water, and free water, respectively. The corresponding peak areas A_21_, A_22_, and A_23_ represent the relative proportions of these three water populations.

**Figure 3:**
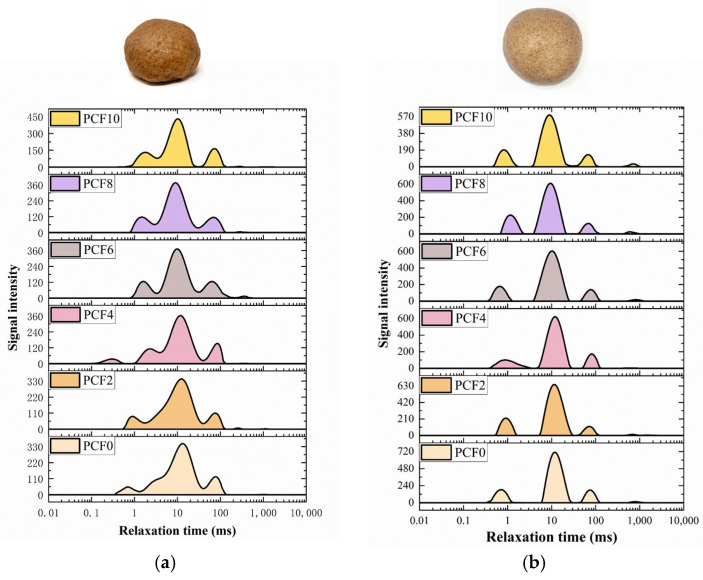
Water distribution in dough with increasing *Polygonatum cyrtonema* flour levels characterized by LF-NMR. (**a**) demonstrates the stacked area plot of moisture distribution in dough; (**b**) illustrates the corresponding plot for steamed bread. PCF0, PCF2, PCF4, PCF6, PCF8, and PCF10 represent samples with 0%, 2%, 4%, 6%, 8%, and 10% (*w*/*w*) *Polygonatum cyrtonema* flour substitution, respectively.

As shown in Tables S3 and S4 and Figure 3, the relaxation times t_21_, t_22_, and t_23_ of steamed bread, both pre- and post-steaming, peaked at a 4% addition of PCF. With an addition level exceeding 4%, the peak area A_21_ reached its peak at 19.46, while A_22_ and A_23_ decreased to their minima. This indicates that PCF facilitated the conversion of some loosely bound water into free water and strongly bound water [27], thereby enhancing water binding to gluten proteins and restricting water molecular mobility. This phenomenon is attributed to two mechanisms: (1) The hydrophilicity of PCF altered the moisture distribution within the dough, promoting tighter binding of water to both starch and gluten proteins; (2) PCF established new cross-links with gluten proteins [28], reinforcing the gluten network and resulting in a more continuous and compact protein-starch matrix, consequently enhancing the dough’s water retention capacity.

### 3.6. Sodium Dodecyl Sulfate–Polyacrylamide Gel Electrophoresis

**Figure 4:**
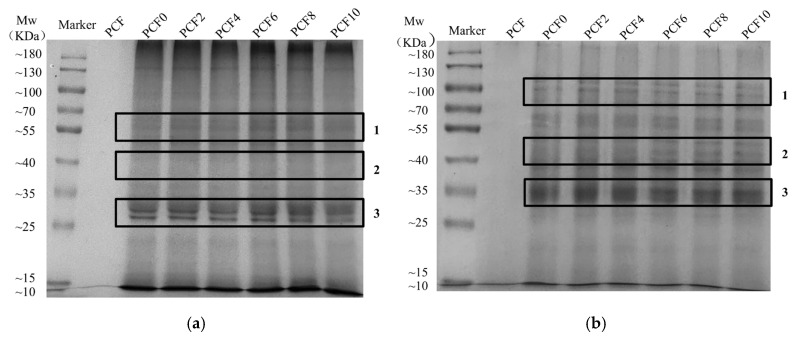
SDS-GE patterns of dough proteins with increasing levels of *Polygonatum cyrtonema* flour, (**a**) non-reducing conditions; (**b**) Reducing conditions. PCF0, PCF2, PCF4, PCF6, PCF8, and PCF10 represent samples with 0%, 2%, 4%, 6%, 8%, and 10% (*w*/*w*) *Polygonatum cyrtonema* flour substitution, respectively.

The molecular weight distribution of proteins in wheat flour dough was analyzed by gel electrophoresis (Figure 4). As shown in Figure 4a, with increasing PCF addition the band intensity in the 55–70 kDa range progressively increased, reaching a maximum at 6% (*w*/*w*) PCF, and then declined with further PCF; meanwhile, in Figure 3a the band intensities in regions 1, 2, and 3 decreased significantly, indicating reduced contents of the corresponding protein fractions. This phenomenon can be attributed to polysaccharides in PCF interacting with the dough protein matrix via hydrogen bonding or hydrophobic interactions; such binding may disrupt interactions between ω-gliadin and other proteins, as well as between γ-gliadin and other gluten protein components, thereby hindering polymeric network formation at the dough stage [29]. Upon PCF addition followed by reduction (Figure 3b), the banding pattern changed markedly: the band intensity in region 1 decreased significantly, whereas those in regions 2 and 3 increased significantly; within the 70–100 kDa molecular-weight range, band resolution progressively improved with increasing PCF level, a change attributable to a more favorable protein composition and enhanced cross-linking capacity of gluten proteins, which promotes the formation of a more stable gluten network structure [29]. Notably, before and after PCF addition, the electrophoretic profiles of wheat dough and its fractions remained overall unchanged, with no new bands observed, suggesting that the effects of PCF on gluten proteins are mediated primarily through non-covalent interactions.

The results indicate that the incorporation of PCF inhibited protein degradation, thereby contributing to the maintenance of network structural integrity within the dough. This effect can be attributed to two primary mechanisms: Firstly, PCF possesses a strong water-holding capacity, which binds water molecules and partially diminishes the hydration and aggregation processes of gluten proteins. Secondly, interactions such as hydrogen bonding and hydrophobic interactions between PCF constituents and proteins play a role in fortifying the gluten network structure.

### 3.7. FT-IR

Protein Fourier transform infrared (FT-IR) spectra encompass the amide I (1600~1700 cm^−1^), amide II, and amide III bands. The amide I band is particularly valuable for characterizing protein secondary structure, as it contains distinct signatures of α-helix, β-sheet, β-turn, and random coil conformations [14].

**Figure 5:**
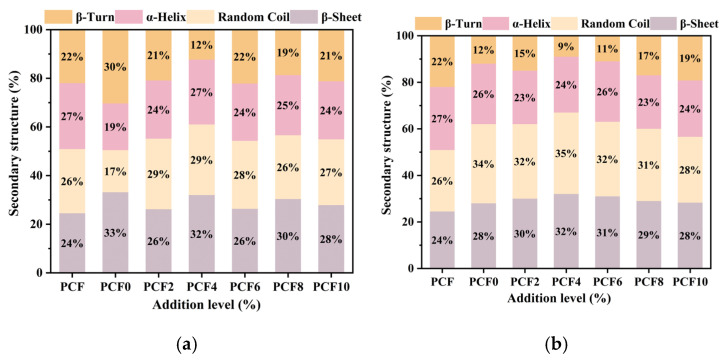
Protein secondary structures of samples with increasing PCF levels: (**a**) dough; (**b**) steamed bread. PCF0, PCF2, PCF4, PCF6, PCF8, and PCF10 represent samples with 0%, 2%, 4%, 6%, 8%, and 10% (*w*/*w*) *Polygonatum cyrtonema* flour substitution, respectively. Means with different superscripts within the same row are significantly different at *p* < 0.05.

As shown in Figure 5a,b, at a 4% PCF addition level the relative content of β-sheet reached a peak of 31.98%, while α-helix was 26.66%, representing a significant 7.47% increase over the control. With increasing PCF, α-helix first rose and then declined, whereas β-turn exhibited the opposite trend (first decreased and then increased); for both secondary structures, the changes were particularly pronounced at the 4% addition level. Previous studies have indicated that β-sheet and β-turn are the dominant secondary-structure components in gluten proteins [14]. In the present work, the increases in β-sheet and α-helix suggest a shift in the protein secondary structure toward a more stable and ordered state. Under the 4% PCF condition, β-sheet and α-helix increased markedly while β-turn decreased; meanwhile, gluten proteins in the dough formed robust inter- and intrachain cross-linked networks via disulfide bonds, hydrogen bonds, and hydrophobic interactions [15], thereby enhancing the stability and structural order of the gluten network. Al-Wraikat et al. [30] reported that a transition from β-turn to β-sheet can strengthen the gluten network; in addition, polysaccharides and guar gum can act synergistically with starch and, through interactions with proteins, improve dough rheology, establishing them as effective dough-quality improvers. These observations are consistent with the conclusions of this study. Composition analysis further shows that PCF is rich in polysaccharides, which can promote the transition of protein secondary structures from disordered to ordered states.

### 3.8. SH and S-S Contents

**Figure 6:**
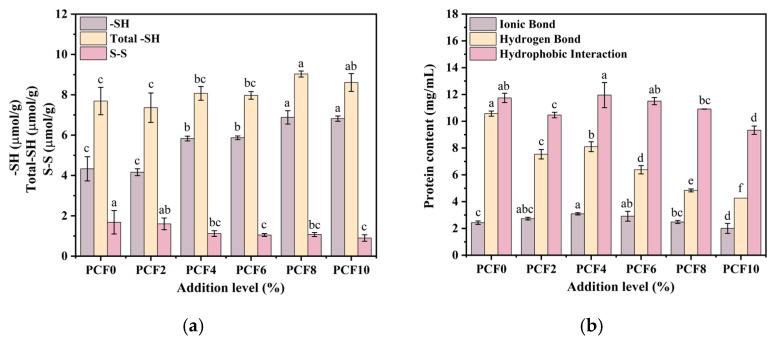
Effects of PCF on Protein Networks: Covalent and Noncovalent Interactions, (**a**) Covalent Interactions; (**b**) Noncovalent Interactions. PCF0, PCF2, PCF4, PCF6, PCF8, and PCF10 represent samples with 0%, 2%, 4%, 6%, 8%, and 10% (*w*/*w*) *Polygonatum cyrtonema* flour substitution, respectively. Means with different superscripts within the same row are significantly different at *p* < 0.05.

The formation and cleavage of disulfide bonds (S-S) typically result in a decrease and increase, respectively, in the content of free sulfhydryl groups (-SH). Consequently, monitoring changes in free -SH content has become a common method to indirectly evaluate modifications in disulfide bonds. To further investigate the impact of PCF addition on gluten proteins in steamed bread, the contents of -SH, total sulfhydryl groups and S-S were analyzed, as outlined in Figure 6a.

The control dough exhibited a free sulfhydryl (–SH) content of 4.33 μmol/g and a disulfide bond (S–S) content of 1.68 μmol/g. With increasing PCF levels, free –SH and total sulfhydryl increased, whereas disulfide bonds decreased. Notably, at 4% PCF, free –SH reached its minimum (5.83 μmol/g) with an S–S content of 1.12 μmol/g. This combination (low free –SH and low S–S) suggests a more balanced gluten network; moreover, enhanced hydrogen bonding can promote gluten aggregation and cross-linking and stabilize peptide chains [31]. At higher PCF levels, the rise in free –SH alongside the decline in disulfide bonds can be ascribed to a dilution effect: substitution with PCF lowers the relative concentration of gluten proteins in the dough, potentially impeding efficient intermolecular disulfide formation. The primary cause is competition for water molecules between PCF components and gluten proteins, reducing gluten hydration and intermolecular interactions. This interpretation aligns with the dough water-holding capacity analysis.

A reduction in S-S content within the dough leads to weakened interactions between gluten protein molecules, leading to reduced dough viscoelasticity and gluten strength, as well as hindering the effective aggregation of gluten proteins. The primary functional component in PCF is its polysaccharide fraction. Research by Yang et al. [32] demonstrated that the addition of fucoidan, a structurally analogous polysaccharide, to dough also reduced disulfide bond content. This observation supports the suggestion made in the current study that PCF polysaccharides may form robust non-covalent interactions with gluten proteins, thereby enhancing network stability at the molecular level. Furthermore, PCF effectively inhibited water migration within the dough system, likely contributing to the preservation of disulfide bond stability, a conclusion in line with the outcomes of water distribution analysis.

### 3.9. Non-Covalent Interactions

Non-covalent interactions—ionic interactions, hydrogen bonding, and hydrophobic interactions—govern the aggregation behavior and three-dimensional structure of gluten. As shown in Figure 6b, ionic interactions in gluten proteins are weaker than hydrogen bonding and hydrophobic interactions, mainly owing to the low abundance of ionizable residues. This compositional feature limits the electrostatic contribution relative to other non-covalent forces within the gluten matrix. PCF markedly shifted the balance of non-covalent interactions: relative to the control, ionic interactions first increased and then decreased with rising PCF, peaking at 3.09 mg/mL at 4% PCF, indicating a relative enhancement at this level and suggesting a transient strengthening of electrostatic associations within the protein matrix. Sodium chloride (NaCl) also promoted hydrogen-bond formation in wheat dough by modulating water activity and water clustering and by strengthening ion–dipole interactions between Na^+^/Cl^−^ and protein polar groups [33], thereby facilitating closer approach of hydrogen-bond donors and acceptors.

Hydrophobic interactions likewise strengthened and then weakened with increasing PCF, reaching 11.95 mg/mL (2% above the control) at 4% before declining. Hydroxyl groups in PCF can foster cross-linking and aggregation of gluten proteins via intermolecular hydrogen bonding, potentially stabilizing or reinforcing hydrophobic associations [34]. The enhancement of these non-covalent interactions, particularly hydrogen bonding and hydrophobic interactions, is corroborated by the results from FT-IR and thiol–disulfide analyses. The FT-IR data showed a significant increase in ordered secondary structures, which are stabilized by these non-covalent forces [35]. Meanwhile, the observed decrease in disulfide bond content indicates that the reinforcement of the gluten network at moderate PCF levels is not achieved through covalent cross-linking, but rather through non-covalent interactions. Taken together, these PCF-induced adjustments to ionic, hydrogen-bonding, and hydrophobic interactions are consistent with a more cohesive yet balanced gluten network near moderate PCF levels, which directly accounts for the improved textural properties and the optimized, uniform pore structure of the resulting steamed bread.

### 3.10. UV–Visible Absorption Spectroscopy

**Figure 7:**
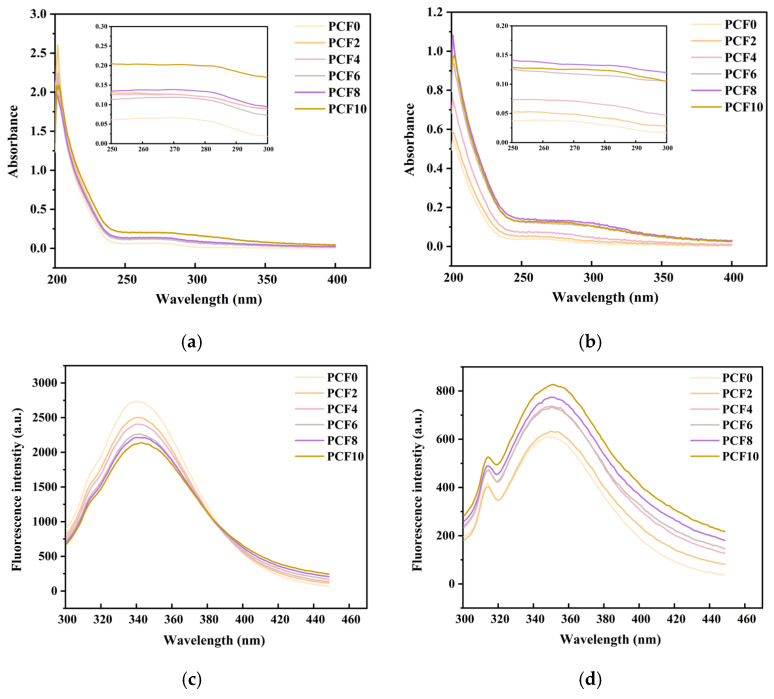
UV and fluorescence spectral analysis of steamed bread with *Polygonatum cyrtonema* flour addition. (**a**) shows the UV spectrum of dough; (**b**) shows the UV spectrum of steamed bread; (**c**) shows the fluorescence spectrum of dough; (**d**) shows the fluorescence spectrum of steamed bread. PCF0, PCF2, PCF4, PCF6, PCF8, and PCF10 represent samples with 0%, 2%, 4%, 6%, 8%, and 10% (*w*/*w*) *Polygonatum cyrtonema* flour substitution, respectively.

UV–visible absorption spectroscopy plays a vital role in investigating the structural transitions of starch and proteins as well as the intermolecular interactions among their components. Variations in the observed absorption intensity can further reflect changes in the secondary and tertiary structures of proteins. As illustrated in Figure 7a,b, a pronounced absorption peak was observed near 265 nm. With the gradual incorporation of PCF into the system, all composite dough samples exhibited a red shift accompanied by an increase in peak intensity. This phenomenon is closely related to the characteristic UV absorption of polysaccharides derived from PCF and wheat flour.

The underlying mechanism can be attributed to the presence of diverse bioactive compounds in PCF, including polysaccharides, steroidal saponins, flavonoids, phenolic acids, and alkaloids. These constituents engage in complex physicochemical interactions within the system, leading to an increased number of reactive residues [36], which significantly influence the UV absorption properties. Moreover, a slight blue shift in the maximum absorption peak of gluten proteins was observed following PCF supplementation, suggesting that PCF altered the interactions among chromophoric groups in wheat proteins. Consequently, modifications occurred in the protein secondary structure, consistent with the findings from secondary structure analyses.

### 3.11. Fluorescence Spectroscopic Analysis

Fluorescence spectroscopy was employed to investigate conformational changes in proteins, as the fluorescence spectrophotometer can record variations in intrinsic fluorophores such as tryptophan residues, thereby reflecting changes in the microenvironment surrounding chromophoric amino acid groups. As presented in Figure 7c,d, the emission maxima of the samples were observed around 340 nm. With the increasing addition of PCF, the overall fluorescence intensity gradually decreased, accompanied by a slight red shift. This indicates that the fluorophores were located in a less hydrophobic environment, suggesting that the hydrophobicity of the system was reduced and the microenvironment surrounding α-amylase became more hydrophilic.

At a PCF substitution level of 10%, the fluorescence intensity reached its maximum, indicating that the amino acid residues were more exposed to hydrophilic regions, resulting in a relatively loose protein conformation. At higher PCF levels, however, the protein structure became more compact, as supported and elucidated by concurrent FT-IR findings, which revealed a significant increase in ordered secondary structures, particularly β-sheets and α-helices, accompanied by a decrease in less ordered motifs. The decline in fluorescence intensity can primarily be attributed to heat treatment during dough processing, which causes partial masking of tryptophan and tyrosine residues due to protein aggregation. Consequently, the hydrophobic peptides formed after thermal treatment tend to be unstable and prone to hydrophobic interactions, resulting in fluorescence quenching [18]. In addition, polyphenols and flavonoids present in PCF may promote non-covalent interactions with proteins, further contributing to fluorescence quenching and modifying surface hydrophobicity, which is consistent with analyses of hydrophobic interactions in non-covalent bonding systems.

### 3.12. Texture Profile and Loaf Specific Volume

**Figure 8:**
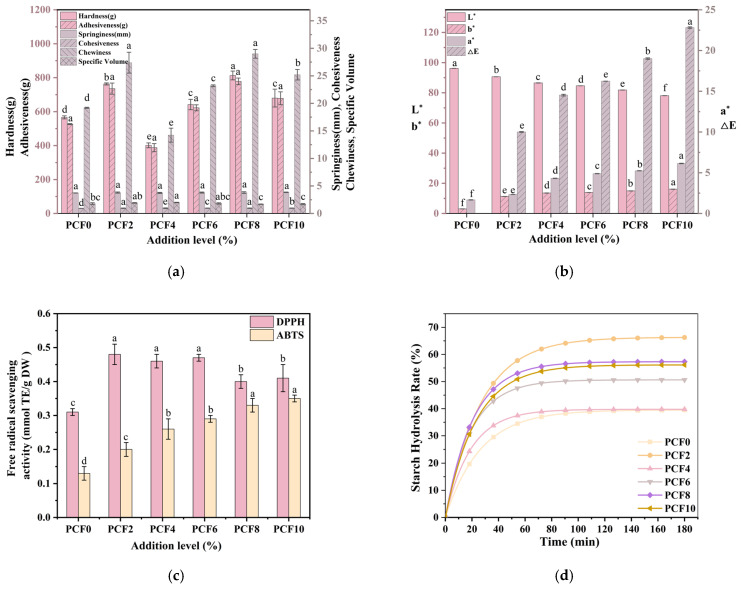
Effects of PCF addition on steamed bread: (**a**) (TPA) parameters and specific volume; (**b**) color difference; (**c**) antioxidant capacity; (**d**) in vitro digestion. PCF0, PCF2, PCF4, PCF6, PCF8, and PCF10 represent samples with 0%, 2%, 4%, 6%, 8%, and 10% (*w*/*w*) *Polygonatum cyrtonema* flour substitution, respectively. Means with different superscripts within the same row are significantly different at *p* < 0.05.

The quality of steamed bread is primarily determined by its textural attributes—springiness, cohesiveness, hardness, gumminess, and chewiness. As indicated in Table S5 and Figure 8a, the incorporation of PCF significantly influences hardness, chewiness, and gumminess. Initially, these attributes increase with rising PCF levels but subsequently decrease. Compared to the control group, steamed bread containing 4% PCF demonstrated reductions of 29% in hardness, 25.8% in chewiness, and 26.3% in gumminess, while simultaneously exhibiting enhanced springiness and cohesiveness. This improvement is attributable to interactions between PCF and gluten proteins, facilitating the development of a more stable and compact gluten network structure, thereby optimizing the textural properties [37]. When the PCF addition exceeds 4%, there is a notable increase in hardness, chewiness, and gumminess. Specifically, at the 6% addition level, there were significant rises of 59.8% in hardness, 63% in chewiness, and 60.6% in gumminess compared to the control. This deterioration in texture may be attributed to the dilution of gluten proteins within the dough matrix by the supplemented PCF, leading to disruption in the development of the gluten network. Consequently, the dough undergoes inadequate expansion during fermentation, diminishing its leavening capacity. As a result, this produces a firmer steamed bread with increased chewiness, negatively impacting its taste and overall quality.

Similar textural changes were also observed in previous studies. Zhang et al. observed that tartary buckwheat flour increased hardness and chewiness while decreasing springiness at high substitution levels due to gluten network disruption [38]. Bilal et al. found that wheatgrass powder affected gluten polymerization and starch gelatinization; moderate substitution improved steamed bread structure, whereas excessive addition produced a denser crumb and greater firmness [39]. Moreover, reviews on coarse cereal–fortified steamed bread and plant-based substitutes for wheat flour consistently indicated that appropriate incorporation of fiber- and phytochemical-rich ingredients enhanced softness and cohesiveness, whereas excessive replacement generally decreased specific volume and increased crumb hardness [40]. These findings collectively supported our conclusion that an appropriate level of PCF acted as a textural improver, while over-fortification caused gluten dilution and led to inferior textural properties.

As presented in Figure 8a, the specific volume of steamed bread increased with the incorporation of PCF, peaking at a 4% addition level. This enhancement suggests that specific components in PCF interact with gluten proteins, stabilizing and strengthening the dough structure during fermentation, thereby improving leavening capacity. Secondly, gliadins crosslink with phenolic compounds through covalent bonds (disulfide bonds) or non-covalent interactions (hydrogen bonding and hydrophobic interactions), reinforcing the gluten network and expanding the specific volume [41]. When the quantity of PCF exceeds 4%, the specific volume diminishes. This decline signifies that an excess of powder disrupts the formation of the gluten network, where hydrophobic interactions from dietary fibers promote an increase in beta-sheet structures while reducing beta-turn configurations in protein secondary structures [42]. Consequently, molecular conformational alterations and gluten aggregation occur, leading to incomplete dough fermentation and a decrease in leavening capacity.

### 3.13. Color Difference

Fundamentally, the pale-yellow color of wheat flour is primarily due to endogenous yellow pigments like lutein and carotenoids, which possess chromophores properties originating from extensive conjugated double-bond systems [43]. Analysis of Table S6 and Figure 8b revealed significant alterations in the chromatic parameters of steamed bread with varying levels of PCF incorporation. Specifically, the L* value (lightness) exhibited a progressive decrease, while the a* (red-green axis) and b* (yellow-blue axis) showed increments. Compared to the control, the 4% PCF formulation demonstrated an 11% reduction in L* value. Moreover, relative to the maximum 10% addition level, a* and b* values increased by 25% and 15%, respectively. This alteration in coloration arises from two key mechanisms: Firstly, PCF contains abundant carotenoids (α-carotene, β-carotene) [44], which contribute to inherent coloration, directly impacting the luminosity of steamed bread during fermentation.

### 3.14. Pore Analysis of Steamed Bread Crumb

**Figure 9:**
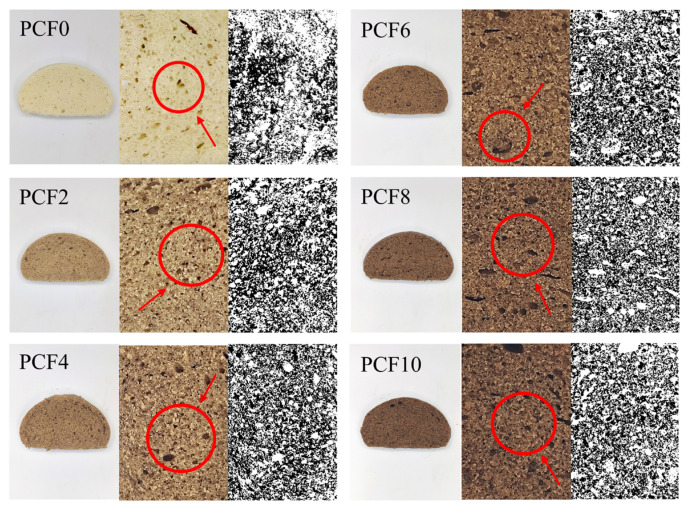
Effect of *Polygonatum cyrtonema* Flour Addition on the Internal Porosity of Steamed Bread. PCF0, PCF2, PCF4, PCF6, PCF8, and PCF10 represent samples with 0%, 2%, 4%, 6%, 8%, and 10% (*w*/*w*) *Polygonatum cyrtonema* flour substitution, respectively.

Using ImageJ, we quantified crumb pore architecture, including pore-size distribution, total porosity, pore number density, and circularity, to index structural uniformity and gas-retention capacity. Figure 9 (PCF0) depicts cross-sections of steamed bread with corresponding internal pore imaging. The black regions in the images represent pore structures mainly formed by CO_2_ gas generated during fermentation. During this phase, yeast metabolizes carbohydrates (e.g., starch and sugars) in the dough to produce CO_2_. The gluten proteins establish an elastic network that captures the CO_2_ gas, preventing its release and facilitating the development of a porous structure within the dough. Larger pores contribute to a softer, more spongy texture, while smaller pores result in a denser, firmer mouthfeel.

As evidenced by the porosity trendline (PCF2), the addition of 2% PCF in steamed bread resulted in a decrease in porosity compared to the control group. The optimized pore structure formed at 4% PCF also provides a direct microstructural explanation for the improved textural properties, namely the reduced hardness and enhanced elasticity. However, beyond 4% additive levels, the crumb structure developed excessive porosity with heterogeneous pore dispersion, concomitant with increased firmness and reduced resilience. This structural alteration corresponds to the increases in hardness and chewiness observed in the texture analysis, and is further corroborated by SEM observations at higher PCF addition levels, which reveal a disrupted and discontinuous gluten network.

### 3.15. Antioxidant Capacity

The incorporation of PCF significantly boosted the radical scavenging capabilities of the steamed bread products. As illustrated in Figure 8c, at appropriate substitution levels, both DPPH and ABTS scavenging rates were markedly increased, indicating that the antioxidant activity was reinforced by polyphenols and polysaccharides. This aligns with the results from fermented black chickpea pasta [19], where fermentation with *Lactiplantibacillus* plantarum led to approximately a 64% increase in DPPH scavenging capacity, a 25% increase in ABTS activity, and a lipid peroxidation inhibition rate approaching 94%. These findings suggest that the augmentation of antioxidant potential in food products can be achieved through either direct supplementation with polysaccharides and polyphenols or the activation of bound phenolics facilitated by fermentation processes.

### 3.16. In Vitro Digestion Analysis

The introduction of PCF resulted in significant modifications to the starch digestibility of the steamed bread products, as depicted in Table 2. From the in vitro starch hydrolysis curves, it is evident that the incorporation of PCF significantly accelerated starch enzymatic hydrolysis, as reflected by a steeper slope in the initial stage and higher final glucose release compared with the control. This trend corresponded to increases in rapidly digestible starch (RDS) and slowly digestible starch (SDS), accompanied by a marked reduction in resistant starch (RS), which declined from approximately 60% in the control to about 34% at the 10% substitution level. The pronounced enhancement in digestibility can be attributed to modifications in starch structure and physicochemical properties. Changes in pasting behavior and elevated gelatinization temperatures indicate that the starch matrix became more susceptible to enzymatic hydrolysis. Furthermore, SEM revealed disruption of the gluten network and greater exposure of starch granules, thereby facilitating enzyme accessibility. These results indicate that PCF addition disrupted the integrity of the starch–protein matrix, enhanced enzyme accessibility to starch granules, and thereby promoted starch hydrolysis, this observation aligns with the results from RVA assessments.

Mechanistically, PCF polysaccharides interacted with gluten proteins to form hydrophilic colloids, improving dough water-holding capacity, elevating gelatinization temperature, and suppressing retrogradation, which collectively rendered starch granules more susceptible to enzymatic degradation [13]. From a nutritional perspective, such changes may increase the postprandial glycemic response, which contrasts sharply with the effects of conventional hydrocolloids, such as guar gum and sodium alginate, that retard starch hydrolysis and maintain RS levels [45]. Although PCF fortification improves dough quality and enhances antioxidant activity, its concomitant reduction of RS suggests a nutritional trade-off. Future product innovation may consider combining PCF with digestion-retarding polysaccharides to simultaneously achieve quality improvement and glycemic control.

The starch hydrolysis kinetics were described using a nonlinear model to obtain the equilibrium hydrolysis concentration (C_∞_) and the hydrolysis rate constant (*K*), thereby constructing the starch digestion curve [46]. As shown in Table 2 and Figure 9, both parameters changed significantly with increasing levels of PCF. The incorporation of PCF markedly increased the C_∞_ value. The K value first decreased and then increased with higher PCF levels, suggesting that moderate PCF addition slowed starch hydrolysis, while excessive substitution might disrupt the gluten–starch matrix, increase porosity, and enhance enzyme diffusion. Appropriate incorporation of PCF could form a less starch-dense network structure and inhibit enzyme adsorption, thereby reducing both C_∞_ and K values, slowing starch digestion, and improving the nutritional quality of the product.

### 3.17. Multiscale Mechanism of Polygonatum cyrtonema Flour in Enhancing Dough Quality

**Figure 10:**
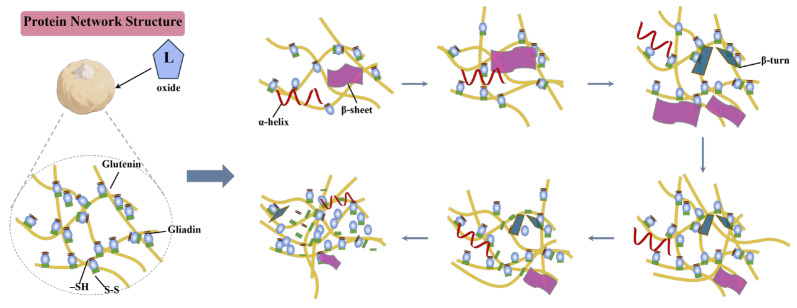
Model of disulfide bond-mediated cross-linking between *Polygonatum cyrtonema* flour and gluten.

This study dives into how PCF works in steamed bread and dough. While we already know a lot about its role in dough, we are now unpacking its mechanism in the final steamed bread product. At a moderate substitution level, PCF polysaccharides interact with gluten proteins mainly through hydrogen bonding and hydrophobic associations, partially replacing disulfide cross-links (Figure 6a). This shift enhances network flexibility while maintaining stability, as supported by FT-IR and thiol–disulfide analyses, the stabilization of protein secondary structures, particularly increased α-helix and β-sheet content, further supports the improved rheological behavior. (Figure 5a,b). PCF also promotes the conversion of loosely bound water into strongly bound states, thereby improving hydration and water retention, as confirmed by LF-NMR results (Figure 3a,b). These molecular changes translate into a denser and more uniform gluten–starch matrix, confirmed by SEM observations (Figure 2). The strengthened structure explains the higher elasticity, cohesiveness, and specific volume observed at the optimal PCF level (Figure 8a). Meanwhile, the polysaccharides in PCF, via hydrogen- or electron-donation, markedly enhance DPPH and ABTS radical scavenging (Figure 8c), which, acting in concert with the denser protein–starch network, endows the product with greater antioxidant potential. With respect to digestive behavior, PCF improves α-amylase accessibility to starch granules, elevating RDS and SDS while significantly lowering RS, and it also alters the digestion kinetics (Figure 8d), indicating a nutritional trade-off of potentially higher postprandial glycemia alongside quality improvement. Combining physicochemical analysis, Figure 10 provides a comprehensive picture of how PCF works in dough and steamed bread [13,27,45].

## 4. Conclusions

This study evaluated the effects of graded PCF substitution on dough hydration, gluten network characteristics, steaming performance, texture, color, microstructure, protein structure and degradation, and in vitro digestibility, as well as antioxidant capacity of steamed bread. The results showed that PCF promoted water binding and stabilizes the gluten network via hydrogen bonding and hydrophobic associations, yielding breads with increased elasticity, cohesiveness, and specific volume while decreasing hardness and chewiness. Microstructural and protein analyses confirmed improved water retention and favorable secondary-structure transitions, collectively reinforcing dough stability. UV–Vis and fluorescence analyses further revealed that PCF strengthened protein–starch interactions and induced a more compact protein conformation, thereby enhancing gluten stability. In addition, PCF enhanced the antioxidant capacity of the final product, providing added functional value. Excessive substitution disrupts gluten integrity and reduces resistant starch content, implying potential drawbacks for glycemic regulation. It should be noted that this study did not examine the shelf-life or storage stability of steamed bread. Future studies are needed to investigate how PCF affects microbial stability, textural evolution, and overall quality during storage. Taken together, these findings indicate that incorporating 4% PCF can deliver technological improvements alongside nutritional enhancement while maintaining overall product quality. After comprehensive evaluation, a PCF substitution at an appropriate level is identified as optimal, providing both improved digestion and nutritional value while offering theoretical support for further PCF applications.

## Figures and Tables

**Table 1:** Effect of *Polygonatum cyrtonema* flour Addition Level on Thermo-mechanical Properties of Dough.

Addition Level (%)	T_O_ (°C)	T_P_ (°C)	T_C_ (°C)	∆H (J/g)
PCF0	58.65 ± 0.45 ^a^	63.19 ± 0.35 ^b^	67.85 ± 0.35 ^d^	5.66 ± 0.77 ^a^
PCF2	58.67 ± 0.11 ^a^	63.28 ± 0.01 ^b^	68.08 ± 0.02 ^cd^	4.75 ± 0.47 ^a^
PCF4	59.01 ± 0.18 ^a^	63.78 ± 0.24 ^a^	68.47 ± 0.04 ^bc^	5.19 ± 0.76 ^a^
PCF6	59.49 ± 0.86 ^a^	64.11 ± 0.02 ^a^	69.32 ± 0.30 ^a^	7.44 ± 2.65 ^a^
PCF8	59.14 ± 0.17 ^a^	64.03 ± 0.10 ^a^	68.90 ± 0.11 ^ab^	5.87 ± 0.12 ^a^
PCF10	59.19 ± 0.21 ^a^	63.96 ± 0.04 ^a^	68.77 ± 0.18 ^b^	5.34 ± 0.05 ^a^

Means with different superscripts within the same row are significantly different at *p* < 0.05. PCF0, PCF2, PCF4, PCF6, PCF8, and PCF10 represent samples with 0%, 2%, 4%, 6%, 8%, and 10% (*w*/*w*) *Polygonatum cyrtonema* flour substitution, respectively.

**Table 2:** Effect of *Polygonatum cyrtonema* flour addition on in vitro starch digestibility of steamed bread.

Addition Level (%)	RDS	SDS	RS	C_∞_/%	K/min^−1^	R^2^
PCF0	21.03 ± 0.54 ^d^	18.48 ± 0.79 ^c^	60.48 ± 1.26 ^a^	39.52 ± 1.26 ^d^	0.04 ± 0.0008 ^d^	0.999
PCF2	25.79 ± 0.17 ^c^	14.00 ± 0.28 ^d^	60.22 ± 0.24 ^a^	39.78 ± 0.24 ^d^	0.05 ± 0.0008 ^a^	0.996
PCF4	32.51 ± 0.71 ^b^	18.12 ± 1.39 ^c^	49.37 ± 0.70 ^b^	50.63 ± 0.70 ^c^	0.05 ± 0.0032 ^a^	0.998
PCF6	35.26 ± 0.62 ^a^	22.06 ± 0.21 ^b^	42.68 ± 0.47 ^c^	57.32 ± 0.47 ^b^	0.05 ± 0.0008 ^b^	0.999
PCF8	32.65 ± 0.66 ^b^	23.49 ± 0.49 ^b^	43.85 ± 0.96 ^c^	56.15 ± 0.96 ^b^	0.04 ± 0.0007 ^c^	0.998
PCF10	35.09 ± 1.50 ^a^	31.23 ± 1.87 ^a^	33.68 ± 2.92 ^d^	66.32 ± 2.92 ^a^	0.04 ± 0.0015 ^d^	0.999

Means with different superscripts within the same row are significantly different at *p* < 0.05. PCF0, PCF2, PCF4, PCF6, PCF8, and PCF10 represent samples with 0%, 2%, 4%, 6%, 8%, and 10% (*w*/*w*) *Polygonatum cyrtonema* flour substitution, respectively.

## Data Availability

The original contributions presented in the study are included in the article/Supplementary Materials, further inquiries can be directed to the corresponding authors.

## Supplementary Materials

The following supporting information can be downloaded at: https://www.mdpi.com/article/10.3390/foods14234116/s1, Table S1: Effect of *Polygonatum cyrtonema* flour on the Farinograph Properties of Composite Flour; Table S2: Effect of *Polygonatum cyrtonema* flour Addition Level on the Pasting Properties of Dough; Table S3: Effect of *Polygonatum cyrtonema* flour Addition Level on Moisture Distribution of Dough; Table S4: Effect of *Polygonatum cyrtonema* flour Addition Level on Moisture Distribution in Chinese Steamed Bread; Table S5. Effect of *Polygonatum cyrtonema* flour Addition on Texture Profile and Specific Volume of Steamed Bread; Table S6. Effects of *Polygonatum cyrtonema* flour Addition on Color Difference of Steamed Bread.

## Abbreviations

The following abbreviations are used in this manuscript:

PCF	*Polygonatum cyrtonema* flour
DSC	Differential Scanning Calorimetry
RVA	Rapid Visco Analyzer
SEM	Scanning Electron Microscopy
FT-IR	Fourier Transform Infrared Spectroscopy
SDS-PAGE	Sodium Dodecyl Sulfate–Polyacrylamide Gel Electrophoresis
LF-NMR	Low-Field Nuclear Magnetic Resonance
DNTB	5,5′-Dithiobis 2-nitrobenzoic acid
DTT	Dithiothreitol
TCA	Trichloroacetic acid
DNS	3,5-dinitrosalicylic acid
UV–Vis	Ultraviolet–Visible Absorption Spectroscopy
DPPH	2,2-diphenyl-1-picrylhydrazyl
ABTS	2,2′-azino-bis (3-ethylbenzothiazoline-6-sulfonic acid)
