# Supplementary material for: Effect of Polygonatum cyrtonema Flour Addition on the Rheological Properties, Gluten Structure Characteristics of the Dough and the In Vitro Digestibility of Steamed Bread
Source: Foods. 2025 Dec 1;14(23):4116. doi: 10.3390/foods14234116 (PMC12692145; doi:10.3390/foods14234116)
Supplement: Supplementary file 1 [file foods-14-04116-s001.zip › foods-3999145-supplementary.pdf]

Table S1. Effect of *Polygonatum cyrtonema* flour on the Farinograph Properties of Composite Flour. PCF0, PCF2, PCF4, PCF6, PCF8, and PCF10 represent samples with 0%, 2%, 4%, 6%, 8%, and 10% (w/w) *Polygonatum cyrtonema* flour substitution, respectively. Means with different superscripts within the same row are significantly different at $p < 0.05$.

Addition Level (%)	PCF0	PCF2	PCF4	PCF6	PCF8	PCF10
**WA (%)**	$60.00±0.00^{e}$	$60.00±0.00^{e}$	$60.40±0.20^{d}$	$61.00±0.00^{c}$	$61.63±0.11^{b}$	$62.33±0.29^{a}$
**C1 Time**	$1.16±0.07^{b}$	$1.29±0.17^{b}$	$1.26±0.15^{b}$	$2.62±0.05^{a}$	$2.54±0.05^{a}$	$2.42±0.18^{a}$
**C1 Torque**	$1.08±0.01^{d}$	$1.09±0.02^{cd}$	$1.14±0.01^{a}$	$1.12±0.01^{ab}$	$1.1±0.01b^{cd}$	$1.11±0.02^{abc}$
**Stability Time**	$6.10±0.00^{a}$	$5.83±0.15^{ab}$	$5.87±0.06^{ab}$	$5.87±0.23^{ab}$	$6.03±0.06^{ab}$	$5.80±0.17^{b}$
**C2 Torque**	$0.37±0.01^{a}$	$0.31±0.01^{b}$	$0.30±0.01^{b}$	$0.27±0.01^{c}$	$0.26±0.01^{cd}$	$0.26±0.01^{cd}$
**C2 Time**	$16.12±0.10^{b}$	$15.58±0.18^{a}$	$15.73±0.11^{a}$	$16.22±0.08^{b}$	$16.13±0.18^{b}$	$16.20±0.08^{b}$
**C3 Torque**	$1.59±0.02^{ab}$	$1.56±0.02^{bc}$	$1.59±0.02^{ab}$	$1.55±0.03^{c}$	$1.60±0.01^{a}$	$1.60±0.01^{a}$
**C4 Torque**	$1.45±0.03^{abc}$	$1.43±0.01^{bc}$	$1.43±0.02^{bc}$	$1.41±0.03^{c}$	$1.46±0.01^{ab}$	$1.48±0.01^{a}$
**C5 Torque**	$2.29±0.06^{a}$	$2.17±0.01^{b}$	$2.22±0.04^{ab}$	$2.14±0.03^{b}$	$2.15±0.03^{b}$	$2.25±0.12^{ab}$

Table S2. Effect of *Polygonatum cyrtonema* flour Addition Level on the Pasting Properties of Dough. PCF0, PCF2, PCF4, PCF6, PCF8, and PCF10 represent samples with 0%, 2%, 4%, 6%, 8%, and 10% (w/w) *Polygonatum cyrtonema* flour substitution, respectively. Means with different superscripts within the same row are significantly different at $p < 0.05$.

Addition Level (%)	Peak viscosity (PV)	Trough viscosity (TV)	Breakdown (BKD)	Final viscosity (FV)	Setback (STB)	Time to peak (min)	Pasting temperature (°C)
**PCF0**	$2291.50±91.22^{a}$	$1936.50±116.67^{a}$	$355.00±25.46^{ab}$	$2963.50±115.26^{a}$	$1027.00±1.41^{a}$	$6.80±0.18^{a}$	$87.95±1.13^{a}$
**PCF2**	$2135.50±9.19^{b}$	$1783.00±25.46^{a}$	$352.50±16.26^{ab}$	$2803.00±33.94^{a}$	$1020.00±8.49^{a}$	$6.57±0.05^{b}$	$87.70±0.57^{a}$
**PCF4**	$1966.50±60.10^{c}$	$1600.00±87.68^{b}$	$366.50±27.58^{ab}$	$2587.00±151.32^{b}$	$987.00±63.64^{a}$	$6.47±0.00^{bc}$	$87.60±0.49^{a}$
**PCF6**	$1892.50±2.12^{c}$	$1577.00±2.83^{b}$	$315.50±4.95^{c}$	$2384.00±21.21^{c}$	$807.00±24.04^{b}$	$6.64±0.05^{ab}$	$88.80±1.20^{a}$
**PCF8**	$1823.00±100.41^{c}$	$1443.50±78.49^{bc}$	$379.50±21.92^{a}$	$2272.50±65.76^{cd}$	$829.00±12.73^{b}$	$6.44±0.05^{bc}$	$88.43±0.60^{a}$
**PCF10**	$1661.00±18.38^{d}$	$1287.50±4.95^{c}$	$396.00±12.73^{a}$	$2119.50±9.19^{d}$	$814.00±12.73^{b}$	$6.32±0.02^{c}$	$88.4±0.21^{a}$

Table S3. Effect of *Polygonatum cyrtonema* flour Addition Level on Moisture Distribution of Dough. PCF0, PCF2, PCF4, PCF6, PCF8, and PCF10 represent samples with 0%, 2%, 4%, 6%, 8%, and 10% (w/w) *Polygonatum cyrtonema* flour substitution, respectively. Means with different superscripts within the same row are significantly different at $p < 0.05$.

Addition Level (%)	T_{21}/ms	A_{21}/%	T_{22}/ms	A_{22}/%	T_{23}/ms	A_{23}/%
**PCF0**	0.69±0.16^{d}	6.66±0.46^{d}	13.36±0.53^{a}	80.54±0.86^{a}	83.26±5.82^{e}	12.80±1.29^{bc}
**PCF2**	0.89±0.07^{d}	9.85±0.50^{c}	12.77±0.89^{a}	78.35±0.30^{a}	350.27±23.82^{cd}	11.80±0.21^{c}
**PCF4**	2.71±0.16^{a}	19.46±0.58^{a}	11.63±0.46^{b}	66.03±1.31^{b}	469.18±34.45^{a}	14.51±1.12^{bc}
**PCF6**	1.56±0.13^{c}	15.40±0.73^{b}	9.89±0.40^{d}	66.24±3.46^{b}	407.92±40.97^{b}	18.35±2.83^{a}
**PCF8**	1.48±0.01^{c}	16.16±0.32^{b}	9.14±0.13^{cd}	68.31±2.57^{b}	387.71±25.11^{bc}	15.53±2.87^{ab}
**PCF10**	1.95±0.32^{b}	18.43±1.03^{a}	10.12±0.40^{c}	65.42±1.20^{b}	340.65±16.38^{d}	16.16±0.53^{ab}

Table S4. Effect of *Polygonatum cyrtonema* flour Addition Level on Moisture Distribution in Chinese Steamed Bread. PCF0, PCF2, PCF4, PCF6, PCF8, and PCF10 represent samples with 0%, 2%, 4%, 6%, 8%, and 10% (w/w) *Polygonatum cyrtonema* flour substitution, respectively. Means with different superscripts within the same row are significantly different at $p < 0.05$.

Addition Level (%)	T_{21}/ms	A_{21}/%	T_{22}/ms	A_{22}/%	T_{23}/ms	A_{23}/%
**PCF0**	11.86±0.03^{a}	15.36±0.44^{d}	11.86±0.03^{a}	71.83±0.5^{b}	878.86±34.81^{a}	12.81±0.53^{a}
**PCF2**	11.63±0.46^{a}	17.63±0.45^{b}	11.63±0.46^{a}	73.36±0.83^{a}	744.54±38.68^{b}	9.10±0.30^{e}
**PCF4**	11.88±0.02^{a}	14.62±0.24^{d}	11.88±0.02^{a}	73.29±0.65^{a}	875.74±23.50^{a}	12.09±0.45^{ab}
**PCF6**	12.17±2.18^{a}	14.83±0.69^{d}	12.17±2.18^{a}	74.20±0.60^{a}	864.98±29.71^{a}	10.97±0.73^{cd}
**PCF8**	9.44±0.37^{b}	19.55±0.29^{a}	9.44±0.37^{b}	70.07±0.41^{c}	712.11±96.88^{b}	10.39±0.39^{d}
**PCF10**	8.84±0.92^{b}	16.42±1.02^{c}	8.84±0.92^{b}	71.78±0.88^{b}	703.71±70.06^{b}	11.81±0.80^{bc}

Table S5. Effect of *Polygonatum cyrtonema* flour Addition on Texture Profile and Specific Volume of Steamed Bread. PCF0, PCF2, PCF4, PCF6, PCF8, and PCF10 represent samples with 0%, 2%, 4%, 6%, 8%, and 10% (w/w) *Polygonatum cyrtonema* flour substitution, respectively. Means with different superscripts within the same row are significantly different at $p < 0.05$.

Addition Level (%)	Hardness(g)	Springiness (mm)	Cohesiveness	Chewiness	Adhesiveness(g)	Specific Volume
**PCF0**	566.33±9.07^{d}	3.72±0.02^{a}	0.94±0.01^{a}	19.20±0.10^{d}	526.67±3.05^{d}	1.79±0.17^{bc}
**PCF2**	763.00±6.08^{b}	3.80±0.11^{a}	0.96±0.04^{a}	27.40±1.90^{a}	735.67±32.72^{a}	1.92±0.07^{ab}
**PCF4**	401.33±14.15^{e}	3.74±0.10^{a}	0.96±0.06^{a}	14.23±1.27^{e}	387.67±24.01^{e}	2.00±0.05^{a}
**PCF6**	641.67±30.75^{c}	3.80±0.10^{a}	0.97±0.02^{a}	23.20±0.17^{c}	622.67±17.67^{c}	1.83±0.12^{abc}
**PCF8**	813.00±26.00^{a}	3.80±0.15^{a}	0.96±0.04^{a}	29.00±0.79^{a}	778.33±19.50a	1.69±0.04^{c}
**PCF10**	680.00±52.56^{c}	3.85±0.05^{a}	0.97±0.03^{a}	25.20±0.95^{b}	678.67±37.90^{b}	1.70±0.11^{c}

Table S6. Effects of *Polygonatum cyrtonema* flour Addition on Color Difference of Steamed Bread. PCF0, PCF2, PCF4, PCF6, PCF8, and PCF10 represent samples with 0%, 2%, 4%, 6%, 8%, and 10% (w/w) *Polygonatum cyrtonema* flour substitution, respectively. Means with different superscripts within the same row are significantly different at $p < 0.05$.

Addition Level (%)	L^*	a^*	b^*	△E
**PCF0**	$96.24±0.01^a$	$1.67±0.02^f$	$3.07±0.04^f$	−
**PCF2**	$90.63±0.02^b$	$2.34±0.01^e$	$11.32±0.02^e$	$10.01±0.05^e$
**PCF4**	$86.52±0.07^c$	$4.32±0.02^d$	$13.54±0.07^d$	$14.53±0.11^d$
**PCF6**	$84.60±0.02^d$	$4.89±0.02^c$	$13.92±0.03^c$	$16.24±0.01^c$
**PCF8**	$81.84±0.03^e$	$5.24±0.02^b$	$14.96±0.07^b$	$19.01±0.09^b$
**PCF10**	$78.06±0.06^f$	$6.14±0.03^a$	$16.11±0.06^a$	$22.82±0.09^a$
